# Identifying Barriers to the Acquisition of Knowledge about Skin Integrity Impairment in Nursing Students: An Educational Intervention

**DOI:** 10.3390/nursrep14020089

**Published:** 2024-05-10

**Authors:** Javier Sánchez-Gálvez, Santiago Martínez-Isasi, Miriam Sánchez-Hernández, Eva Vegue-Parra, Tamara Rafaela Yacobis-Cervantes, Francisco Mateo-Ramírez, Daniel Fernández-García

**Affiliations:** 1 Doctoral Programme in Health, Disability, Dependence and Welfare, University of León, 24071 León, Spain; jsanchez29@ucam.edu; 2 Faculty of Nursing, UCAM—Universidad Católica de Murcia, Campus de Cartagena, 30310 Cartagena, Spain; msanchez6@ucam.edu (M.S.-H.); evegue@ucam.edu (E.V.-P.); tryacobis@ucam.edu (T.R.Y.-C.); fmateo@ucam.edu (F.M.-R.); 3 Simulation, Life Support, and Intensive Care Research Unit (SICRUS), Health Research Institute of Santiago de Compostela (IDIS), 15706 Santiago de Compostela, Spain; 4 Primary Care Interventions to Prevent Maternal and Child Chronic Diseases of Perinatal and Developmental Origin (RICORS) (RD21/0012/0025), Carlos III Health Institute, 28029 Madrid, Spain; 5 Clinical Nursing, Emergencies, Simulation and Teaching Innovation Research Group (CLINURSID), Department of Psychiatry, Radiology, Public Health, Nursing, and Medicine, University of Santiago de Compostela, 15704 Santiago de Compostela, Spain; 6 Health Research Nursing Group (GREIS), Department of Nursing and Physiotherapy, University of León, 24071 León, Spain; dferg@unileon.es

**Keywords:** nursing, knowledge, nursing education, nursing student, wounds and injuries, wound healing

## Abstract

Background: Wound healing competence is implied in the nursing profession, but there is no standardized content regulation for wound care in university curricula. The primary objective of this study was to identify the barriers to the acquisition of knowledge about skin integrity impairment. Methods: A quasi-experimental pre-test and post-test study with an ad hoc questionnaire involved 304 students (control: 165; intervention: 139) from June to July 2023. A 10-h educational intervention focused on skin integrity assessment and treatment was conducted. Results: The control group, scoring 17 ± 0.22 out of a maximum of 61, achieved a significantly lower final test score (*p* < 0.001) compared to the wound care educational intervention group, with the pre-test group scoring 30 ± 0.76 and the post-test group scoring 43 ± 0.61. The educational intervention in wound care program improved nursing students’ knowledge of prevention, assessment/diagnosis, treatment, lower limb wounds, and wound bed preparation by replacing the number of "Don’t know" answers in the post-test group with correct answers. Conclusions: The barriers identified to the acquisition of knowledge about skin integrity impairment in nursing studies are the following: the transversality of teaching, the teaching and evaluation system, and the variability in the training of professionals and teachers in charge of their education. The educational intervention can be used to consolidate knowledge and to enhance students’ self-confidence in caring for patients with wounds.

## 1. Introduction

Wounds are disruptions in the continuity of the skin caused by various factors, and their origins can be attributed to different pathologies [1]. Wounds can be categorized as either acute or chronic based on their healing process, evolution over time, and the presence of pathogenic anomalies that may impede the healing process.

Dependence-related skin lesions and pressure ulcers have a prevalence of 11.61% in Spain, with the majority being treated in primary care services and accounting for 35% to 65% of patient visits [2,3]. Essentially, lower limb ulcers and neuroischemic ulcers comprise the greatest workload in nursing, both in primary care and in multidisciplinary units specializing in wounds [4,5].

To achieve competence in wounding healing, European nurses require extensive knowledge and the adoption of new, evidence-based solutions to enhance their expertise, ensuring the delivery of quality care to patients with wounds [6]. Although this competence is implied in the profession, the Resolution of 20 October 2020 of the General Direction of Public Health, which validates the “Guide for the indication, use and authorization of dispensing medicines subject to medical prescription by the nurses of: wounds”, is the one document where the role of the nurse in the care of skin injuries, ulcers, or wounds is indicated [7].

Knowledge about wound treatment, academic training in skin integrity, and positive behavior towards patients should form the basis of an efficient understanding of how to assist patients with wounds [8]. However, there is no standardized content regulation for wound care in the university curricula. It is often left to the individual discretion of professors whether to include this topic in their courses. In many universities, wound care training is typically integrated across various subjects during the degree program. In Spain, only three universities offer an optional course specifically dedicated to this topic [9].

Several studies aim to understand the teaching load regarding wound care in nursing undergraduate programs, revealing significant variations [10]. Generally, students begin to receive education in the first year, covering topics such as skin histology and its physiology, as well as potential problems. The remaining education (the assessment, care, and treatment of dermatological conditions, acute wounds, and chronic wounds) is distributed throughout the rest of the degree program across different subjects, which may pose a challenge for professionals during their university studies [11].

Furthermore, in the European Credit Transfer and Accumulation System [ECTS], aspects focusing on impaired skin integrity are few, comprising two ECTS, corresponding to about 50 h of student work, but insufficient in the total balance of the degree [12]. It is assumed that, as a result of this training during their undergraduate degree, students should possess knowledge regarding skin integrity impairments. They should also be able to apply this knowledge to their work in a professional manner. Nurses are not able to reach an appropriate knowledge level in wound care, have a lack of comprehension of wound care, and feel insecure in performing wound care due to educational deficiency focusing on this topic. Considering this educational context, it is not surprising that wound care based on traditional practices or the personal preferences of professors continues to be carried out today, ignoring the new scientific evidence that is available [13].

Education on wound care beyond the nursing undergraduate degree in level Spain is based on continuing education courses, training provided by commercial laboratories, numerous university expert courses in wound care for patients, and a university Master’s degree [14]. Some studies link the low level of knowledge about chronic wound management among postgraduate nurses in various care areas with the fragmentation and lack of objectives in the undergraduate curriculum [15].

Educational interventions have taken place based on previously identified training needs after a process of deep reflection [16]. Training interventions for nurse practitioners in wound assessment have been shown to help improve patient safety and benefit patients [17,18]. Interventions conducted on students have been shown to develop clinical skills about wound care [19,20].

The primary objective was to identify the barriers to the acquisition of knowledge about skin integrity impairment in nursing students from a university in Spain. The secondary objective was to analyze the effectiveness of an educational intervention about wound healing in nursing students.

## 2. Materials and Methods

### 2.1. Design

A quasi-experimental study was designed as a pre-test and post-test with an ad hoc questionnaire. The educational intervention was carried out over 10 hours, in which the knowledge about the assessment and treatment of damage to skin integrity was presented to pre-grade nurse students in a university located in the southeast of Spain. The study was completed between June and July of 2023.

### 2.2. Participants

The study population was 304 students, of which 165 made up the control group (CG) and made up 139 the intervention group (IG) during the period from June to July 2023.

### 2.3. Inclusion and Exclusion Criteria

The inclusion criteria for the control group were final year students of a Bachelor’s degree in Nursing who agreed to complete the questionnaire and signed an informed consent form.

The inclusion criteria for the intervention group were students of a Bachelor’s degree in Nursing who were not part of the control group, wished to participate in training on wound care, and agreed to complete the questionnaire and signed an informed consent form.

The exclusion criteria for both groups were students of other Bachelor’s degree programs than Nursing and students who did not complete the questionnaire in its entirety or did not sign the informed consent form.

### 2.4. Measures

Dependent variables were evaluated through 61 items with direct questions on these topics and 4 more items on the theoretical and practical training received. These variables each had three levels, corresponding to the three possible responses for each item: Yes, No, and Don’t know/Don’t answer.

These variables were dependence-related skin lesion prevention (items 1 to 15), chronic wound treatment (items 16 to 28), and pressure ulcer assessment/diagnosis (items 29 to 39). Knowledge about lower limb ulcers was evaluated in items 40 to 49, and specific aspects about wound bed preparation were assessed in items 50 to 61.

The independent variables were the following:

Demographics included age (a continuous variable that adopted values between 22 and 54); sex (dichotomous nominal variable); studies prior to the Degree in Nursing (nominal variable with 5 levels); and the course in which they are enrolled (nominal variable with 4 levels).

Training intervention was a nominal variable that adopted 2 values (intervention group and control group). The intervention group received 10 hours of training on the prevention, assessment/diagnosis, and treatment of wounds, lower limb ulcers, and wound management/wound healing (described below).

### 2.5. Procedure

An ad hoc questionnaire was developed for data collection, consisting of 61 closed-ended questions with 3 response options, of which only one was valid (ANNEX I). The creation of the questionnaire consisted of two phases.

In the first phase, after a review of the literature, 61 questions were selected in relation to wound assessment and care. This was complemented by a second questionnaire composed of 4 items. The initial questionnaire was sent as a pilot test to 5 professional nurses. In the pilot test, considerations were made regarding the language used, the format of the answers, and the relevant characteristics of the study population. The questionnaire was composed of questions with the answers “Yes, No, Don’t Know/Don’t Answer”.

Breaking down the questionnaire, the sources of the questions are as follows:

- Items 1–39: Extracted and adapted from the evidence recommendations of the Practical Guide for Pressure Ulcers and the Practical Guide for Moisture Associated Skin Damages from the Collection of Practical Guides of Wounds of the Servizo Galego de Saúde [21,22], which aggregates several evidence-based clinical practice guidelines [23,24].
- Items 40–49: Extracted and adapted from the evidence recommendations of the Practical Guide for Ulcers of the Lower Limb from the Collection of Practical Guides of Wounds of the Servizo Galego de Saúde [25], which aggregates several evidence-based clinical practice guidelines [26,27,28]
- Items 50–61: Extracted and adapted from the study “Degree of knowledge of nursing in primary health care on the cure in wet environment and the use of dressings” [29].
- Regarding the thematic content of the questionnaire, it is divided into seven sections:
- Initial questions (4): Personal and professional sociodemographic data of the participants (age, sex, studies prior to the Degree in Nursing, course in which they are enrolled);
- Items 1 to 15: Dependence-related skin lesion (DRSL) prevention;
- Items 16 to 28: Chronic wound treatment;
- Items 29 to 39: Pressure ulcer assessment/diagnosis;
- Items 40 to 49: Lower limb ulcer assessment/diagnosis;
- Items 50 to 61: Wound bed preparation;
- Items 62–65: Questions about wound training (theoretical and practical) received prior to completing the questionnaire.

The final test score was calculated as the sum of correct answers (from item 1 to item 61). A low level of knowledge was considered when between 1 and 20 correct answers were obtained, a medium level was considered when between 21 and 40 correct answers were obtained, and a high level was considered when between 41 and 61 correct answers were obtained.

Second phase: Based on the responses and suggestions received, the final questionnaire was prepared. The dissemination of the questionnaire to the participants was anonymous and voluntary, self-completed, and in online format (Google Form^®^). The questionnaire was distributed through the internal lists of the Faculty of Nursing, with a collection period of one month. Fifteen days were allocated as an ordinary period, followed by an additional fifteen days of reinforcement (reminder) in case the necessary sample size was not reached by the end of the first period.

Simultaneously, the intervention group was formed by offering an elective course to nursing degree students who voluntarily enrolled in the course. This was structured into 5 theoretical online sessions, each lasting 2 hours, where the topics were presented through lectures with visual support, including explanatory images, photographs, and videos. During and after each session, there were discussions regarding the practical application of the theoretical content, the clinical experiences of the students, and related questions. Students were evaluated through the resolution of a practical case.

The theoretical content was distributed as follows:

- Session 1: The skin: structure, function, and healing process; skin integrity impairment; classification of wounds; moist wound healing; products and treatments.
- Session 2: Wound management: dependence-related skin lesions; pressure ulcers; etiology, assessment, classification, treatment, and prevention.
- Session 3: Lower limb ulcers: etiology, assessment, classification, differential diagnosis, treatment, and prevention.
- Session 4: Surgical wounds, incised–contused wounds, and burns: etiology, assessment, classification, treatment and referral criteria.
- Session 5: Periwound skin: physiology, assessment, classification, and treatment; periwound skin as a predictor of healing.

The aim of this educational intervention was to equip students with the following competencies:

- Demonstrate possession and comprehension of knowledge regarding skin integrity impairment and relate this to the knowledge acquired during their undergraduate education.
- Apply this knowledge to their work in a professional manner and obtain the necessary competencies to do so.
- Gather and interpret relevant data to make judgments including reflection on the care of patients with ulcers or wounds.

They were invited to complete the same ad hoc questionnaire as an initial assessment and again 10 days after the end of the intervention to check whether the intervention had been effective.

### 2.6. Sample Size

The sample size calculation to compare two proportions of 17% (CG) to 43% (GI) and assuming an alpha of 0.01 indicated that 111participants per group would be necessary to have a statistical power of 95%.

### 2.7. Data Analysis

Data are presented as the total score of tests or as the percentage of students that answer correct, incorrect, or Don’t know/Don’t answer to the test’s questions. The significance level (*p*) was fixed at 0.05 (* *p* < 0.05; ** *p* < 0.01; *** *p* < 0.001).

The normality of the data regarding the total score of the tests was previously assessed using a Shapiro–Wilk test and the homogeneity of variance was also verified using the Levene test. For non-normally distributed data, a non-parametric Mann–Whitney U test was used to compare scores between the control group and the pre-test and post-test groups. Differences within the pre-test and post-test groups were assessed using the Wilcoxon signed-rank test.

To determine differences between the answers of the control and pre-test or post-test groups, respectively, χ^2^ tests were performed, while to assess differences between pre-test and post-test groups, the McNemar test was performed. Statistical analyses were conducted using SPSS 27.0 (SPSS, Chicago, IL, USA).

### 2.8. Ethics

The questionnaires were anonymous, and no information that could identify participants was collected. Each participant declared that the provided data were true by checking a box and gave their consent for the processing of their data and responses for the purpose of conducting the study and the subsequent dissemination of results.

This study was conducted in accordance with the Declaration of Helsinki and approved by the UCAM Research Ethics Committee with code CE062303.

## 3. Results

### 3.1. Demographics

Female students were predominant in the student samples (74.36%), with ages varying between 20 and 54 years old, and 70.1% of students gained access through higher education studies. A total of 63% of the sample consisted of students who had just finished their degree studies, with 55.9% of them constituting the control group. Final-year students comprised 44.1% of the intervention group, while 32.4% of students were from other academic years (Table 1).

Table 2 shows the students’ perceptions of their training. Regarding the topic of whether they had received training on the prevention and diagnosis of wounds, the control group presented a higher percentage (90.3%) of students who received training than the intervention group (80.6%). The percentage of students who received training on wound treatment was slightly lower, comprising 81.2% of the control group and 71.2% of the intervention group. The greatest differences were observed regarding the topic of whether students had worked with wounds, where 95.2% of the control group declared that they had worked with wounds during their training, while in the intervention group, only 50.4% had worked with patients with wounds. Finally, very few students stated that they had participated in wound research, with a slightly higher percentage in the control group (19.4%) than in the intervention group (13.7%).

### 3.2. Educational Intervention in Wound Care Program Increases the Knowledge of Nursing Students

The students in the control group (final year students) achieved a final test score lower than that of students who participated in the educational intervention in wound care group, and this difference was statistically significant (*p* < 0.001 in both groups with control) (Table 3). Within the educational intervention in wound care groups, a significant increase in the final test score was observed when students took the test after the educational intervention in wound care (*p* < 0.001 between pre- and post-test groups) (Table 3).

### 3.3. The Educational Intervention in Wound Care Program Improves the Knowledge of Nursing Students about Prevention, Assessment/Diagnosis and Treatment

All groups showed an increase in their knowledge of the “prevention of wounds” than the “assessment/diagnosis or treatment of wounds”.

The control group achieved 71.76% correct answers about prevention (Table 4); the next best scores were for assessment and diagnosis, where the correct answers’ percentage was 59.67% (Table 4). A lower level in the control group was obtained for questions about the treatment of wounds, being 55%. The percentage of correct answers was 71% (Table 4).

The results regarding correct answers from the educational intervention in wound care group, before intervention (pre-test group), were observed, highlighting that the knowledge about prevention (64.89% correct) (Table 4) was superior to knowledge about assessment/diagnosis (47.87% correct) (Table 4) and treatment (46.38% correct), and the latter categories were very similar (Table 4). Moreover, these results were slightly lower than those of the control group in terms of prevention for both assessment/diagnosis and treatment (Table 4), although the difference in correct answers between the control group and the pre-test group was only significant regarding questions about prevention (*p* = 0.015) (Table 4).

After the educational intervention in wound care (post-test group), the understanding of these three topics improved; this trend continued further, with the percentage of correct answers being slightly higher for prevention (80.34% correct) (Table 4), followed by assessment (68.61% correct) (Table 4) and, finally, treatment (62.37% correct) (Table 4). Despite this increase, the difference was not significant between the control group and the post-test group in each of the three topics (*p* = 0.198, *p* = 0.669, and *p* = 0.837, respectively) (Table 4).

The incorrect answer percentage was lower for questions about prevention, varying between 16.26 and 19.27% in the three groups, and was lower in the post-test group, even though the differences were not significant between either the control and pre-test groups (*p* = 0.884) or the control and post-test groups (*p* = 0.418) (Table 4). In the evaluation–diagnosis questions, the incorrect answer percentage was 28.25% in the post-test group, 32.11% in the pre-test group, and 32.62% in the control group (Table 4). Finally, the greatest percentages of incorrect answers in the three groups were for treatment questions, which were between 30.44% (pre-test group) and 33.80% (control group); nevertheless, the difference between the control group and the pre-test and post-test groups was not significant (Table 4).

The percentage of “I don’t know/I don’t answer” answers is where the more significant differences are observed between the three groups for the three topics (Table 4). There was a greater percentage of students that answered “I don’t know/I don’t answer” in the pre-test group than the control group, with significant differences (Table 4). Furthermore, the percentage of students that answered “I don’t know/I don’t answer” in the post-test group was significantly different to the control group for the three topics (*p* = 0.026, *p* = 0.031 and *p* = 0.001, respectively) (Table 4). When data are observed for each topic, the percentage of students who answered “I don’t know/I don’t answer” to questions about prevention decreased after the intervention, at 3.41% for the post-test group, while in the control group, the percentage was 8.97% (*p* = 0.001 between the control and post-test groups), with these differences being significant (Table 4).

Students in the control group that answered “I don’t know/I don’t answer” to the question about assessment/diagnosis comprised 7.71% of the total, displaying a statistically significant difference between the educational intervention in wound care groups (the pre-test and post-test groups (both *p* = 0.001)) (Table 4).

Finally, a statistically significant reduction was observed in the number of students who responded “I don’t know/I don’t answer” to treatment-related questions after the educational intervention in wound care (Table 4). For the topic of treatment, the control group exhibited significant differences, with 10.49% of the students answering “I don’t know/I don’t answer” in both educational intervention in wound care groups: the pre-test and post-test groups (*p* = 0.038 and *p* = 0.031, respectively) (Table 4).

When the results after intervention were compared, the results regarding correct answers increased for the three topics, while incorrect answers and “I Don’t Know/I Don’t answer” decreased in the three topics (Table 5). The percentage of correct answers from the post-test group was significantly higher than the pre-test group in three topics: prevention, assessment/diagnosis, and treatment (Table 5). Likewise, the percentage of Don’t Know/Don’t answer responses was significantly lower in the post-test group than in the pre-test group (Table 5). Nevertheless, in the post-test group, the percentage of incorrect answers was only significantly lower in the topic of assessment/diagnosis, while the topics of prevention and treatment did not show a statistically significant difference from the pre-test group (Table 5).

### 3.4. The Educational Intervention in Wound Care Program Increases the Understanding of Nursing Students about Specific Wounds and Specific Wound Treatments

The data showed that specific knowledge about wounds, such as lower limb ulcers or healing in wet environments, was lower than knowledge of the three general topics (prevention, assessment/diagnosis, and treatment) (Table 6). The percentage of correct answers was minor, at 50% in both the control group and the group before the educational intervention in wound care (pre-test group) (Table 6). The students that correctly answered questions about lower limb ulcers in the control group comprised 47.70 of the total, while this percentage was 35.62% in the pre-test group, although these differences were not significant (*p* = 0.082) (Table 6). On the other hand, in questions about healing in wet environments, the percentage of correct answers in the pre-test group was 20.08%, significantly lower than the percentage of correct answers in the control group, at 35.91% (*p* = 0.003) (Table 6).

After the educational intervention in wound care, the post-test group showed a significant increase in their understanding of both lower limb ulcers and healing in wet environments. However, these increases in the post-test group were not significant with respect to the control group (both lower limb ulcers, *p* = 0.405, and healing in wet environments, *p* = 0.435) (Table 6).

The percentage of incorrect answers did not show significant changes between questions about lower limb ulcers (*p* = 0.363 for control and pre-test groups; *p* = 0.88 for control and post-test groups), with 21.15% in the pre-test group, 22.36% in the control group, and 25.47% in the post-test group (Table 6). Looking at the data for incorrect answers about healing in wet environments, the percentage values were bigger than those obtained for the other topics in the three groups, reaching a higher percentage of incorrect answers in the post-test group, at 41.37%, followed by the control group, at 39.49%, and the pre-test group, at 36.36%, although only the difference in incorrect answers reported by the control and pre-test groups was significant (*p* = 0.033) (Table 6).

The greatest changes were observed when the students answered “I don’t know/I don’t answer” (Table 6). For questions about lower limb ulcers, the control group and pre-test group did not present significant differences (*p* = 0.06), with the percentage of incorrect answers being larger in the pre-test group (42.23%) than in the control group (29.49%) (Table 6). However, after the educational intervention in wound care, this percentage was significantly reduced in the post-test group (7.70%) with respect to the control (*p* < 0.001) (Table 6).

Finally, for questions related to healing in wet environments, the percentage of students in the pre-test group that answered “I don’t know/I don’t answer” was significantly higher than that in the control (*p* < 0.001), at 43.59% and 24.60%, respectively (Table 6). The differences between the post-test and control groups were also significant for the topic of healing in wet environments (*p* < 0.001) (Table 6).

The results were also better after the intervention in topics about lower limb ulcers and wound bed preparation (Table 7). However, this improvement was not observed for the percentage of incorrect answers as it was not statistically significant between the pre-test and post-test groups (Table 7). When the numbers of correct answers from the pre-test and post-test groups were compared, the increase in two topics was statistically significant (Table 7). Likewise, the percentage of “I Don´t Know/I Don´t answer” responses was significantly lower in the post-test group than in the pre-test group (Table 7).

## 4. Discussion

The main areas that revealed an educational gap were wound bed preparation and lower limb ulcers. Correct responses related to chronic wound assessment, diagnosis, and treatment were close to 50%, with only knowledge regarding the prevention of dependence-related skin lesions surpassing this threshold. The data for this student sample are similar to the data presented by the National Institute of Statistics (2022), which indicate that 84.1% of the nurses in Spain are female. This study has a higher percentage of female nurses (78%) than male nurses (22%) [30]

The control group was composed of students that finished their nursing grade the same year. This characteristic allowed us to evaluate the final level of knowledge that was obtained by the students after finishing the grade. The final questions of the questionnaire provided us with a better understanding of the knowledge acquired beyond the students’ academic degree.

Despite the control group having finished their grade, our results suggest that they had a lower level of knowledge than the pre-test group, which could be due to the evaluation system only focusing on memorization and passing exams quickly, decreasing students’ reasoning and reflection about the subject they studied [31,32].

Other studies indicate that an effect of interventions is that students have greater certainty in answering questions [33,34]. The incorporation of web-supported education alongside conventional teaching positively enhanced the learning outcomes of nursing students [35]. The result of our intervention showed that the students’ certainty was increased as the post-test group had a significantly reduced number of “Don´t know/Don´t answer” responses and an increased rate of the correct answers, suggesting a consolidation of their acquired knowledge [36,37,38]. The certitude of the theoretical knowledge about skin integrity damage leads to better clinical practice, as patient assessments and the healing process will be carried out continuously, and this certitude is associated with increased decision-making for treatment [39,40,41].

On the other hand, the theoretical content about other wounds, such as lower limb ulcers, is very little in this grade, comprising 6 h in the classroom, and could be increased via clinical simulations and external training in hospitals. Our intervention comprised 2 h on this topic, leading to improvements in the understanding about lower limb ulcers’ physiopathology and care, diminishing the number of “Don’t know/Don’t answer” responses in the post-test group. Improvements in the teaching strategy on this topic would allow students to overcome barriers to knowledge [42].

When we observed the incorrect answers across all topics, the results showed that there were no significant differences in all three groups. However, the correct answers increased significantly because the number of “Don´t know/Don´t answer” responses was significantly reduced, suggesting an improvement in understanding due to a consolidation of the acquired knowledge.

The principal difficulties found regarding students´ ability to acquire knowledge about impaired skin integrity were as follows:

Cross-disciplinarity without critical assessment: During the four years of the grade, teaching about skin integrity damage is imparted across different subjects or forms of training without a consecutive or chronological order. This could lead to students facing difficulties in relating the concepts acquired in different subjects, such as microbiology, physiology, pharmacology, clinic subjects, etc., when they have to assist a patient with wounds. In the Nursing Degree program in Spain, the basic theoretical teaching about the assessment and management of wounds is between 0.5 and 2 ECTS, which is very light compared to the 6 ECTS recommended by the European Wound Management Association [10,43].

One suggestion to solve this problem of a shortage of hours is that a specific and obligatory subject about this topic is incorporated into grade studies, following the advice of the European Wound Management Association with a total of six ECTS, comprising four theoretical ECTS and two training ECTS (with small learning groups), with the objective of improving the care of people with wounds [44].

Currently, the evaluation and teaching systems are focused on the student memorizing the topic in a short time without tools for relating or remembering. Different strategies have been attempted to innovate and improve teaching to solve these problems [45]. The use of virtual reality [46], flipped classrooms [47], peer role-play, and clinic simulations could allow students to obtain cohesion in their knowledge of different subjects [48,49], although these strategies are underused in university training about skin integrity damage [50].

Students´ learning could be negatively affected by disjointed teaching, according to different nursing professionals, especially regarding their knowledge of impaired skin integrity, at both the assistance level and educational level [13]. The different criteria for these topics, excluding critical thinking, that are transmitted to students during their theoretical and training education lead to a negative impact on care, leading to longer recovery times, higher costs, and, finally, a reduced quality of life in patients due to appropriate wound healing being hindered [51].

## 5. Limitations

In this study, it was not possible to randomize the sample because it required students completing their degree to form the control group and students who did not fill out the questionnaire to form the intervention group, resulting in a convenience sample bias. The subjectivity of the responses may be a limitation of this study. The use of a non-validated ad hoc questionnaire does not enable comparisons with results from other studies. As this study is based on a limited number of people, we suggest that a large-scale multicentric study should be conducted.

## 6. Conclusions

The main identified barriers to the acquisition of knowledge about wound care during undergraduate nursing studies are the cross-disciplinary nature of the education provided, the teaching and evaluation system, and the variability in the training of professionals and teachers responsible for students’ education.

This study demonstrates how the intervention group significantly improved their knowledge compared to the control group. Students’ access to information as a unit rather than as a cross-sectional contribution facilitates their learning and their ability to understand the relationship between concepts. The intervention facilitated the consolidation of knowledge, instilling self-confidence in students to make informed decisions in the care of patients with wounds.

There is a clear need to implement standardized undergraduate teaching and assessment of nursing students’ knowledge of skin integrity impairment. This would enable comparative studies between universities and the design of concrete training interventions.

## Figures and Tables

**Table 1:** Demographic characteristics.

Variables		CG		IG		Total	
		(*n* = 165)	%	(*n* = 139)	%	(*n* = 304)	%
Sex	Masculine	50	30.3	28	20.1	78	25.7
	Feminine	115	69.7	111	79.9	226	74.3
Age	≤22	89	53.9	56	40.3	145	47.7
	23–27	42	25.5	49	35.3	91	29.9
	28–32	15	9.1	16	11.5	31	10.2
	≥32	19	11.5	18	12.9	37	12.2
Academic year	4th	165	100	28	20.1	193	63.5
	3rd	0	0	35	25.2	35	11.5
	2nd	0	0	47	33.8	47	15.5
	1st	0	0	29	20.9	29	9.5
Previous studies	High-school degree	119	72.1	94	67.6	213	70.1
	Associate degree	24	14.5	15	10.8	39	12.8
	University	19	11.5	14	10.1	33	10.9
	Others	3	1.8	16	11.5	19	6.3

**Table 2:** Percentages of students’ answers to questions about training and working with wounds.

	% Yes Answers		% No Answer		% “I Don’t Know I Don’t Answer” Response	
	CG	IG	CG	IG	CG	IG
Training in prevention and diagnosis	90.3%	80.6%	8.48%	17.9%	1.2%	1.4%
Wound treatment training	81.2%	71.2%	14.6%	23.7%	3.6%	5.0%
Work with wounds	95.2%	50.4%	4.2%	48.2%	0.6%	1.4%
Work on research	19.4%	13.7%	80.6%	83.5%	0.0%	2.9%

**Table 3:** Final test scores of the control or educational intervention in wound care group.

	CG	IG	*p*-Value
Final score pre-test	17 ± 0.22	30 ± 0.76	**<0.001**
Final score post-test		43 ± 0.61	**<0.001**
*p*-value		**<0.001**	

Data represent means ± S.E.M. of the scores of students. *p*-values marked in bold show statistically significant differences between both groups.

**Table 4:** Comparative students’ percentage answers to questions about prevention, assessment/diagnosis and treatment in control group with pre-test group and control with post-test group.

	% Correct Answer			% Incorrect Answer			% “I Don’t Know/I Don’t Answer” Response		
	CG	GI	*p*-Value	CG	GI	*p*-Value	CG	GI	*p*-Value
	**Pre-test**								
**Prevention**	71.76%	64.89%	**0.015**	19.27%	18.47%	0.884	8.97%	16.64%	0.074
**Assessment and Diagnosis**	55.71%	47.87%	0.082	33.80%	32.11%	0.974	10.49%	20.01%	**0.001**
**Treatment**	59.67%	46.38%	0.065	32.62%	30.44%	0.608	7.71%	23.19%	**0.038**
	**Post-test**								
**Prevention**	71.76%	80.34%	0.198	19.27%	16.26%	0.418	8.97%	3.41%	**0.026**
**Assessment and Diagnosis**	55.71%	62.37%	0.669	33.80%	32.54%	0.844	10.49%	5.09%	**0.001**
**Treatment**	59.67%	68.61%	0.837	32.62%	28.25%	0.664	7.71%	3.14%	**0.031**

Data represent the percentage of correct, incorrect, or I don’t know/I don’t answer in control, pre-test and post-test groups. *p*-values marked in bold show statistically significant differences between both groups.

**Table 5:** Percentage of responses of students in the intervention group to questions on prevention, evaluation–diagnosis and treatment.

	% Correct Answer			% Incorrect Answer			% “I Don’t Know/I Don’t Answer” Response		
	Pre-Test	Post-Test	*p*-Value	Pre-Test	Post-Test	*p*-Value	Pre-Test	Post-Test	*p*-Value
Prevention	64.89%	80.34%	**0.001**	18.47%	16.26%	0.07	16.64%	3.41%	**0.001**
Assessment and Diagnosis	47.87%	62.37%	**0.003**	32.11%	32.54%	**0.02**	20.01%	5.09%	**0.003**
Treatment	46.38%	68.61%	**0.003**	30.44%	28.25%	0.42	23.19%	3.14%	**0.001**

Data represent the percentage of correct, incorrect, or “I Don’t Know/I Don’t Answer” answers in pre-test and post-test groups. *p*-values marked in bold show statistically significant differences between both groups.

**Table 6:** Comparative percentages of students’ answers to questions about lower limb ulcers and wound bed preparation in control group with pre-test group and control with post-test group.

	% Answer Correct			% Answer Incorrect			% Answer Don’t Know/Don’t Answer		
	CG	GI	*p*-Value	CG	GI	*p*-Value	CG	GI	*p*-Value
	Pre-test								
Lower limb ulcers	47.70%	36.62%	0.082	22.36%	21.15%	0.363	29.94%	42.23%	0.059
Wound bed preparation	35.91%	20.08%	**0.003**	39.49%	36.33%	0.033	24.60%	43.59%	**0.007**
	Post-test								
Lower limb ulcers	47.70%	66.83%	0.405	22.36%	25.47%	0.88	29.94%	7.70%	**<0.001**
Wound bed preparation	35.91%	48.44%	0.435	39.49%	41.37%	0.248	24.60%	10.19%	**<0.001**

Data represent the percentage of correct, incorrect, or “I Don’t Know/I Don’t Answer” responses in the control, pre-test, and post-test groups. *p*-values marked in bold show statistically significant differences between both groups.

**Table 7:** Percentage of students’ answers to questions about lower limb ulcers and wound bed preparation.

	% Correct Answer			% Incorrect Answer			% “I Don’t Know/I Don’t Answer” Response		
	Pre-Test	Post-Test	*p*-Value	Pre-Test	Post-Test	*p*-Value	Pre-Test	Post-Test	*p*-Value
Lower limb ulcers	36.62%	66.83%	**0.005**	21.15%	25.47%	0.07	42.23%	7.70%	**0.005**
Wound bed preparation	20.08%	48.44%	**0.002**	36.33%	41.37%	0.18	43.59%	10.19%	**0.002**

Data represent the percentage of correct, incorrect, or “I Don’t Know/I Don’t Answer” responses in pre-test and post-test groups. *p*-values marked in bold show statistically significant differences between both groups.

## Data Availability

The data used to support the findings of this study are available from the corresponding author upon request.

## Public Involvement Statement

There was no public involvement in this research.

## Guidelines and Standards Statement

This manuscript was drafted against the CONSORT 2010 Statement: updated guidelines for reporting parallel group randomized trials [52].
